# Mms1 is an assistant for regulating G-quadruplex DNA structures

**DOI:** 10.1007/s00294-017-0773-9

**Published:** 2017-11-02

**Authors:** Eike Schwindt, Katrin Paeschke

**Affiliations:** European Research Institute for the Biology of Ageing (ERIBA), University Medical Center Groningen, University of Groningen, 9713 AV Groningen, The Netherlands

**Keywords:** DNA secondary structures, Pif1 helicase, Genome stability, *S. cerevisiae*

## Abstract

The preservation of genome stability is fundamental for every cell. Genomic integrity is constantly challenged. Among those challenges are also non-canonical nucleic acid structures. In recent years, scientists became aware of the impact of G-quadruplex (G4) structures on genome stability. It has been shown that folded G4-DNA structures cause changes in the cell, such as transcriptional up/down-regulation, replication stalling, or enhanced genome instability. Multiple helicases have been identified to regulate G4 structures and by this preserve genome stability. Interestingly, although these helicases are mostly ubiquitous expressed, they show specificity for G4 regulation in certain cellular processes (e.g., DNA replication). To this date, it is not clear how this process and target specificity of helicases are achieved. Recently, Mms1, an ubiquitin ligase complex protein, was identified as a novel G4-DNA-binding protein that supports genome stability by aiding Pif1 helicase binding to these regions. In this perspective review, we discuss the question if G4-DNA interacting proteins are fundamental for helicase function and specificity at G4-DNA structures.

## Introduction

Secondary DNA structures, such as G-quadruplex (G4) structures, are hypothesized to hamper DNA and RNA-related processes due to their high thermodynamic stability and by this challenging genome integrity (reviewed in Tarsounas and Tijsterman 2013). G4 structures are guanine-rich, four-stranded structures that can form within nucleic acids, if a defined nucleotide sequence, called G4 motif is present (Gellert et al. 1962).

Multiple analyses demonstrate that, once formed, G4 structures positively and negatively influence biological processes (reviewed in Bochman et al. 2012; Maizels and Gray 2013), such as DNA replication (Foulk et al. 2015; Valton et al. 2014), transcription (Law et al. 2010; Siddiqui-Jain et al. 2002), and translation (Morris et al. 2010). Currently, they are discussed as a molecular fine-tuning mechanism of the cell that influences specific processes (David et al. 2016; Nguyen et al. 2014; Tang et al. 2016). However, due to their stability, the formation and unfolding is predicted to be slow (Hazel et al. 2004; Lane et al. 2008). If G4 structures are a regulatory tool in the cell, fast and efficient regulation mechanisms are required. To this date, multiple helicases have been identified that can unwind G4 structure in vitro (Sauer and Paeschke 2017). Interestingly, in vivo experiments support the idea that most G4 structure-regulating helicases are specific for a given cellular process. For example, FANCJ, the helicase linked to the human disorder Fanconi anemia, as well as Pif1 helicases are shown to regulate G4 formation during DNA replication (Castillo Bosch et al. 2014; Paeschke et al. 2011, 2013; Piazza et al. 2017; Ribeyre et al. 2009; Wu and Spies 2016). In the absence of Pif1 in yeast, or FANCJ in humans, deletion or mutations occur at G4 motifs and genome instability accumulates. Therefore, it is not surprising that mutations in many G4-regulating helicases are linked to human genetic disorders (reviewed in Maizels 2006; Mendoza et al. 2016).

In addition to helicases, other machineries/proteins have been shown to support the disruption of formed G4 structures such as translesion synthesis proteins, homologous recombination (HR), or telomerase holoenzyme (Edwards et al. 2014; Paeschke et al. 2008; van Kregten and Tijsterman 2014). How these proteins act specifically at G4 structures or how they communicate with helicases is not clear, yet. Although the regulation of G4 structures is very efficient and G4 structures have an impact on all stages of the cell cycle, it seems that the proteins/helicases that regulate G4 disruption are highly process specific. For example, Pif1 helicase has been shown to act specifically at formed G4 structures during DNA replication, whereas the RecQ helicases are proposed to act at G4 structures during telomere maintenance (reviewed in Sauer and Paeschke in press). To this date, it is not completely understood how this specificity is achieved; one hypothesis is that G4 structure-interacting proteins support helicase binding and thereby increase helicase specificity. In recent years, many scientists performed global approaches to identify novel G4 interacting proteins (reviewed in Mishra et al. 2016).

### Mms1 is a novel factor supporting Pif1 helicase binding to G4s

Using a pull-down-based approach, we identified in yeast, among known G4 interacting proteins, Mms1 as a novel G4-interacting protein (Wanzek et al. 2017). We demonstrated that Mms1 acts together with Rtt101 and Mms22 and forms an ubiquitin ligase complex, Rtt101^Mms1/Mms22^ (Zaidi et al. 2008). All three components are required for replication fork progression after treating cells with the alkylating agent methyl methanesulfonate (MMS), which causes replicative stress (Luke et al. 2006; Vaisica et al. 2011). In line with our observations, the previous studies have shown that Mms1 and Mms22 are required for HR at stalled forks but not at HO-induced double-strand-break (DSB) sites (Duro et al. 2008; Zaidi et al. 2008).

The replication machinery slows at G4 structures (Anand et al. 2012; Valton and Prioleau 2016), especially if G4 structures are not unwound by a DNA helicase, e.g., Pif1 (Paeschke et al. 2011). We speculated that in *S cerevisiae*, the ubiquitin ligase Rtt101^Mms1/Mms22^ is also involved in replication fork progression at G4 motifs. Chromatin immunoprecipitation (ChIP) experiments coupled with deep-sequencing analyses supported the hypothesis. Analyses revealed that the guanine-rich Mms1-binding motif can form “relaxed” G4 structures in vitro. These “relaxed” G4 motifs form less stable G4 structures, because they only harbor two guanines per G-tract (GGN_1–8_GGN_1–8_GGN_1–8_GG) as compared to three or more in canonical G4 structures (e.g.: GGGN_1–7_GGGN_1–7_GGGN_1–7_GGG) (Gellert et al. 1962). Interestingly, “relaxed” G4 motifs that are bound by Mms1 are located on the lagging strand template for DNA replication (Wanzek et al. 2017). In addition, detailed analyses of Mms1 target regions showed that they are also targeted by Pif1 (Wanzek et al. 2017).

Previously, it was shown that Pif1, a 5′–3′ DNA helicase, is a robust unwinder of G4 structures in vitro (Ribeyre et al. 2009). Using two different methods in yeast, it was demonstrated that in Pif1 mutant cells (*pif1-m2*), replication slows at G4 motifs (Paeschke et al. 2011). Furthermore, different genetic analyses revealed that Pif1 preserves genome stability at G4 motifs, and accordingly, deletions and/or mutations occur at these motifs in the absence of Pif1 (Lopes et al. 2011; Paeschke et al. 2013). This led to the model that Pif1 unwinds G4 structures in vivo and by this supports replication fork progression and genome stability. Although these studies nicely support each other, they also harbor controversial aspects. Under one experimental setting, Pif1 acts equally well on G4 motifs on both strands of the replication fork machinery. In contrast, studies that used a repetitive G4 motif from a human minisatellite (CEB1) in yeast showed that Pif1 acts only at G4 motifs located on the leading strand template for DNA replication (Lopes et al. 2011; Paeschke et al. 2013).

**Fig. 1:**
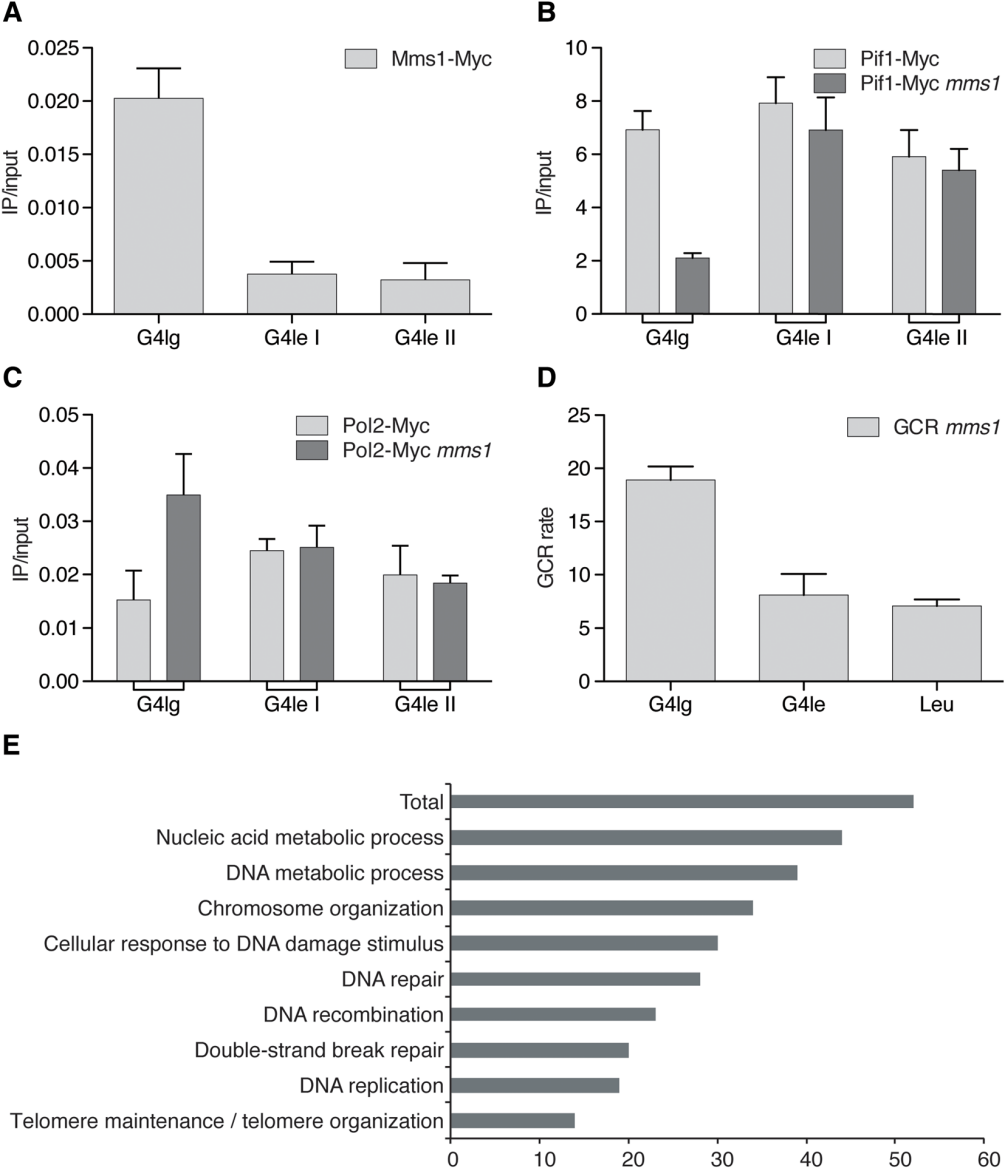
Mms1 does not support Pif1 function at all targets. **a** Mms1 does not bind to G4 motifs on the leading strand. ChIP experiments, using endogenous Myc-tagged Mms1 at specific loci, as well as genome wide analyses (Wanzek et al. 2017) revealed that Mms1 does not bind to G4 motifs on the leading strand (G4lg). Here and in all subsequent ChIP experiments, plotted IP/input values are mean values ± standard deviation (SD) of three biological replicates. **b** Pif1 binding is not affected at G4 motifs on the leading strand. ChIP experiments using endogenously Myc-tagged Pif1 were performed in WT (light grey) and *mms1Δ* (dark grey) cells. Pif1 binding was reduced at a specific G4 motif on the lagging strand (G4lg) in *mms1Δ* cells (Wanzek et al. 2017), whereas binding was unaffected at G4 motifs on the leading strand (G4le). **c** Replication fork progression is not dependent on Mms1 at all G4 motifs. DNA Pol2 binding to G4 motifs was analyzed by ChIP using endogenous Myc-tagged Pol2 and qPCR in WT (light grey) and *mms1Δ* (dark grey) cells. As previously (Wanzek et al. 2017), DNA Pol2 binding was enhanced in the absence of Mms1 at G4 motifs on the lagging strand (G4lg), but no difference in DNA Pol2 binding was observed at other G4 motifs, located on the leading strand template (G4le). **d** G4 motifs on the leading strand did not increase the GCR rate in *mms1*Δ cells. The GCR rate was determined in wild-type and *mms1*Δ cells. Plotted data are normalized to WT. All inserts were integrated using the LEU2 marker. Inserts are: G4 motif on the lagging strand template (G4lg) (Wanzek et al. 2017), G4 motif on the leading strand template (G4le), as well as the *LEU2* marker alone. **e** Analysis of the 52 joined genetic and physical interaction of Pif1 and Mms1 by processes

The newest published results shed some light into this discussion. They revealed that Mms1 supports Pif1 binding to specific G4 structures located on the lagging strand (Wanzek et al. 2017). Without Mms1, Pif1 binding is reduced, replication slows at G4 motifs, and G4-dependent genome instability is observed. The effects are similar to effects reported in Pif1 mutant cells (Paeschke et al. 2011, 2013; Piazza et al. 2012). Mms1 does not bind to G4 motifs on the leading strand (Fig. 1a). Expectedly, in the absence of Mms1, Pif1 binding to G4s on the leading strand was unaffected (Fig. 1b). In addition, no replication pausing and consequently no increased genome instability by GCR were observed, if the G4 motifs was located on the leading strand (Fig. 1c, d). Interaction network analyses showed a significant overlap between Pif1 and Mms1 (50 proteins interacted genetically and 2 proteins physically with both proteins, Fig. 1e). These results provide information in the discussion, whether G4 motifs on the leading, on the lagging strand, or both cause genome instability. It is probably depending on the substrate, the genetic surrounding, and consequently the associated proteins which determine the fate of the G4 motif and Pif1. In practice, this means that repeats (e.g., CEB1) are targeted by a different set of proteins than other G4 motifs. Furthermore, the current data indicate that specific G4 motifs on the lagging strand are targeted by Mms1 and consequently Pif1, but provide no information about other G4 motifs on both leading and lagging strand. In addition, because Mms1 acts here without the Ubiquitin complex, which is an unusual setting for Mms1, it is likely that other proteins are involved in this G4 regulation process. These data and consequent speculation exhibit that disruption of G4 structures is not as trivial as originally anticipated. Presumably a whole complex of proteins acts together to control G4 unwinding at different genomic settings.

G4 structures are discussed to play a positive role in different biological processes such as transcriptional regulation, where they supposed to serve as a loading scaffold for proteins (Bochman et al. 2012). Multiple analyses have now shown that G4 motifs fold into G4 structures in vivo (Biffi et al. 2013, 2014; Paeschke et al. 2005; Schaffitzel et al. 2001) and that G4 motifs are conserved throughout evolution (Capra et al. 2010; Nakken et al. 2009). This and other data suggest that G4 structures have a biological function and fold on purpose and not by chance. Even though it is not fully understood yet, how G4 structures form efficiently and specifically, recent data imply that Mms1 plays a role during G4 structure formation, because it binds to G4 motifs throughout the whole cell cycle (Wanzek et al. 2017) where it has the potential to recruit other G4 modulators.

According to the Saccharomyces Database (SGD) and further publications, there are with Est1, Est2, Rap1, Mre11, Rad50, Xrs2, Pif1, Rrm3, Sgs1, Yku70, Rif1, Hop1, Kem1, Stm1, and Sub1; at least 15 proteins known to bind to DNA G4 structures in *S. cerevisiae* (Cherry et al. 2012; Fisher et al. 2004; Kanoh et al. 2015; Liu and Gilbert 1994; Lopez et al. 2017; Muniyappa et al. 2000; Sun et al. 1999; Van Dyke et al. 2004). Comparing genetic and physical interaction maps of those proteins shows that 5 proteins are genetically or physically linked to Mms1 in *S. cerevisiae*. A genetic/physical interaction does not proof a link, but a potential idea on how to address G4 regulation and unfolding in the near future. The identification of proteins that aid helicase binding and function will be interesting by itself but will also be beneficial for medical aspects. G4 structures are discussed as novel therapeutically targets to regulate biological processes (Balasubramanian and Neidle 2009; Bates et al. 2017; Cogoi and Xodo 2016). The current research focuses on chemical components (ligands) that induce or stabilize G4 structures *in vivo* (Salgado et al. 2015). The aim is to influence/block specific processes by inducing/stabilizing G4 structures. Due to the high number of G4 motifs in the cell over 500/300,000 in *S. cerevisiae*/human (Capra et al. 2010; Chambers et al. 2015; Huppert and Balasubramanian 2005), the current challenge is the specificity of these ligands to certain and not all G4s. Targeting the functional helicases at the right spatiotemporal moment will be an additional difficulty due to their multifunctional nature. However, a promising future strategy to gain specificity could be to target the aiding proteins instead of a multifunctional helicase.
